# Synthesis of Organic–Inorganic Hybrid Perovskite/MOF Composites from Pb–MOF Using a Mechanochemical Method

**DOI:** 10.3390/molecules28135021

**Published:** 2023-06-27

**Authors:** Xinlan Han, Jinhua Li, Siyu Tao, Guowei Dou, Sanawar Mansur, Xinqian Zhang

**Affiliations:** College of Chemistry and Chemical Engineering, Xinjiang Agricultural University, Urumqi 830052, China; 320203326@xjau.edu.cn (X.H.);

**Keywords:** Pb–MOF, organic–inorganic hybrid perovskite/MOF composites, mechanochemical method, optical properties

## Abstract

The specific structure and diverse properties of hybrid organic–inorganic perovskite materials make them suitable for use in photovoltaic and sensing fields. In this study, environmentally stable organic–inorganic hybrid perovskite luminescent materials using Pb–MOF as a particular lead source were prepared using a mechanochemical method. Based on the fluorescence intensity of the MAPbBr_3_/MOF composite, the mechanized chemical preparation conditions of Pb–MOF were optimized using response surface methodology. Then, the morphological characteristics of the MAPbBr_3_/MOF composite at different stages were analyzed using electron microscopy to explore its transformation and growth process. Furthermore, the composite form of MAPbBr_3_ with Pb–MOF was studied using XRD and XPS, and the approximate content of MAPbBr_3_ in the composite material was calculated. Benefiting from the increase in reaction sites generated from the crush of Pb–MOF during mechanical grinding, more MAPbBr_3_ was generated with a particle size of approximately 5.2 nm, although the morphology of the composite was significantly different from the initial Pb–MOF. Optimal performance of MAPbBr_3_/MOF was obtained from Pb–MOF prepared under solvent-free conditions, with a milling time of 30 min, milling frequency of 30 Hz and ball–material of 35:1. It was also confirmed that the mechanochemical method had a good universality in preparing organic–inorganic hybrid perovskite/MOF composites.

## 1. Introduction

The specific structure and diversified properties of hybrid organic–inorganic perovskite materials enable their extensive application in the photovoltaic and sensing fields [1,2,3,4]. Inside the hybrid halide perovskite (ABX_3_), although there is a strong electrostatic potential between the ammonium molecule (A^+^) and framework three-dimensional inorganic network (BX_3_^−^), the volatility of the ammonium components still undermines the long-term air instability [5]. Therefore, these materials are prone to losing their structural integrity under ambient atmosphere, which seriously affects their optical properties [6,7,8] and hinders their further development. MOF is a particular material composed of metal ions or clusters held together by organic ligands, which have a stable structure and different chemical functions [9,10]. Li Liang et al. prepared a MAPbBr_3_@Pb–MOF composite through liquid-phase reactions using ultrasonically synthesized Pb–MOF. This composite was utilized as an anticounterfeiting substance for the encryption and decryption of information. Meanwhile, the Pb^2+^ within Pb–MOF can serve as the lead source during the formation of perovskite particles. This in situ preparation of perovskite@MOF composites results in materials with luminescent properties. During this process, the Pb–MOF provides a specific growth environment for the perovskite, which reduces the aggregation of perovskite particles [11]. Tsai et al. demonstrate that perovskite nanocrystals stabilized in Pb–MOF thin films make bright and stable LEDs. The perovskite nanocrystals in MOF thin films can maintain photoluminescence and electroluminescence against continuous ultraviolet irradiation, heat and electrical stress. As a result, the stability of the perovskite material can be effectively enhanced [12].

Mechanochemical synthesis is a rapid, environmentally friendly method based on a chemical reaction triggered by mechanical milling, which can use just a small amount of solvent or no solvent at all, and consequently is conducive to large-scale production and process sustainability while reducing toxicity [13,14]. James et al. synthesized Zn–MOF-74 employing a mechanochemical method to react ZnO with 2,5-dihydroxyterephthalic acid. This solvent-free process offers a rapid and efficient approach to MOF synthesis, eliminating the need for large amounts of solvents [15]. Grätzel et al. proposed an efficient room-temperature mechano-synthesis of the hybrid organic–inorganic perovskite MAPbI_3_ with well-defined structure and composition. The solar cell devices fabricated from these particles demonstrated obvious advantage over the routine solution based synthetic routes in terms of device performance [16]. Maji et al. reported a stable organic–inorganic hybrid perovskite@MOF composite materials (MAPbBr_3_@MA–M(HCOO)_3_) synthesis through a solvent-free mechanochemical method by grinding PbBr_2_ with MA–M(HCOO)_3_), in which the MA within the MOF serves as the organic component source for MAPbBr_3_ perovskite quantum dots [17]. Synthesizing Pb–MOF and converting it into perovskite/MOF composites through a mechanochemical method is expected to provide a green and efficient approach to preparing environmentally stable organic–inorganic hybrid perovskite luminescent materials.

In this study, Pb–MOF was prepared through single-factor experiments and orthogonal experiments initially. Based on the fluorescence intensity of the MAPbBr_3_/MOF composite formed by the transformation (ball milling of Pb–MOF with MABr), the mechanized chemical preparation conditions of Pb–MOF were optimized using response surface methodology. Then, the morphological characteristics of the MAPbBr_3_/MOF composite at different stages were analyzed using electron microscopy to explore its transformation and growth process. Furthermore, the composite form of MAPbBr_3_ with Pb–MOF was studied using XRD and XPS, and the approximate content of MAPbBr_3_ in the composite material was calculated. Finally, the general applicability of mechanized chemical methods in the preparation of hybrid perovskite/MOF composites is discussed.

## 2. Results and Discussion

### 2.1. Preparation of Pb–MOF

**Figure 1:**
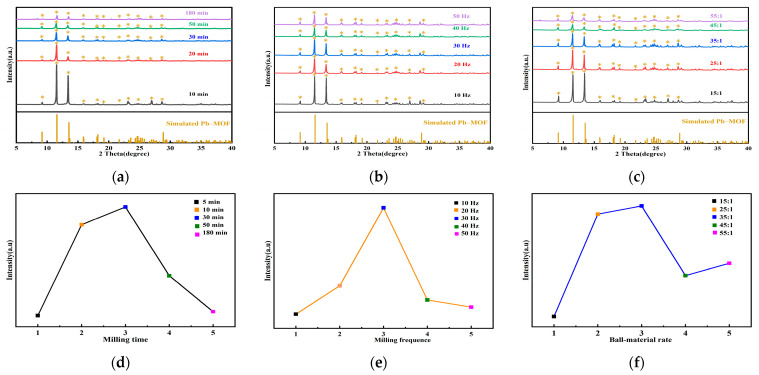
Impact of mechanochemical conditions on XRD of Pb–MOF (**a**) milling time; (**b**) milling frequency; (**c**) ball–material rate; impact of mechanochemical conditions on fluorescence intensity of MAPbBr_3_/MOF composites (**d**) milling time; (**e**) milling frequency; (**f**) ball–material rate; the asterisks in subfigures (**a**–**c**) represent the main simulated characteristic diffraction peaks of Pb–MOF.

A series of Pb–MOFs were prepared by mechanochemical methods, and the conditions for the mechanochemical preparation of Pb–MOF were screened according to the changes in fluorescence intensity of the transformed MAPbBr_3_/MOF composites. A univariate analysis of variance was first performed on the mechanochemical conditions and it was found that Pb–MOF could be synthesized under all of the different mechanochemical conditions, which can be determined by the XRD pattern of the Pb–MOFs (Figure 1a–c, the diffraction peaks at 9.1°, 11.5°, 13.4°, 15.9°, 18.2°, 19.2°, 21.7°, 23.5°, 24.7°, 26.9°, and 29.1° matched well with the simulated XRD pattern), although the fluorescence intensity of the corresponding MAPbBr_3_/MOF compositesvaried obviously. The fluorescence intensity of the composite increased as the milling time of Pb–MOF was extended (within a time frame of 10–30 min). However, when the milling time of Pb–MOF exceeded 50–80 min, a decrease in the fluorescence intensity of the composites was observed (Figure 1d). Additionally, the fluorescence intensity of the composites enhanced gradually with the increase in milling frequency (10–30 Hz). When the milling frequency of Pb–MOF became excessively high (40–50 Hz), the fluorescence intensity of the composites decreased (Figure 1e). Furthermore, when the ball-to-material ratio was set between 20:1–35:1, the fluorescence intensity of the composites was higher (Figure 1f). The reason may be that the growth of MAPbBr_3_ was hampered by the larger Pb–MOF crystal sizes (higher XRD peak intensity) which resulted from the shorter ball milling time, lower frequency, and smaller ball-to-material ratio. Whereas an excessively long ball milling time, ultrahigh frequency, and too large ball-to-material ratio yielded relatively smaller Pb–MOF (lower XRD peak intensity), resulting in excessive and rapid growth of MAPbBr_3_, thereby affecting the luminescent properties of MAPbBr_3_/MOF.

Based on the univariate experiment, the orthogonal experiments of three factors and three levels (Table S1) were designed using Design-Expert 8.0 under Box–Behnken mode, and 17 experiments were performed to optimize the mechanical–chemical conditions of Pb–MOF (Table S2). The fluorescence data from the experiments were subjected to response surface regression model variance analysis, and the quadratic multiple regression equations of A, B and C with the MAPbBr_3_/MOF composites fluorescence intensity (Y) were obtained as follows:

Y = 425 − 34.13A − 72.75B − 42.38C + 60.5AB − 1.75AC − 8.5BC − 169.88A^2^ − 54.62B^2^ − 26.38C^2^

The F value of the model was 34.14, and *p* value was less than 0.001, which indicated the significance of the model. Additionally, the F value of the lack-of-fit item was 2.5 and the *p* value was greater than 0.05, indicating that the lack-of-fit was not significant (Table S3). Among the factors, the order of the F value sizes was factor B (milling frequency) > factor C (ball–material ratio) > factor A (milling time), illustrating that the milling frequency had the greatest impact on the final product’s fluorescence intensity in the mechanochemical method. Error regression analysis was performed on the relevant data, and the calculation results showed that R^2^ and Adj R^2^ were both greater than 0.9, and the difference between Adj R^2^ and PredR^2^ was less than 0.2 (Table S4). The nonlinear relationship between milling frequency, milling time, ball–material ratio and fluorescence brightness was quite significant, indicating the good fit, strong significance and high reliability of the model, which demonstrates its applicability in predicting and analyzing the experimental results.

**Figure 2:**
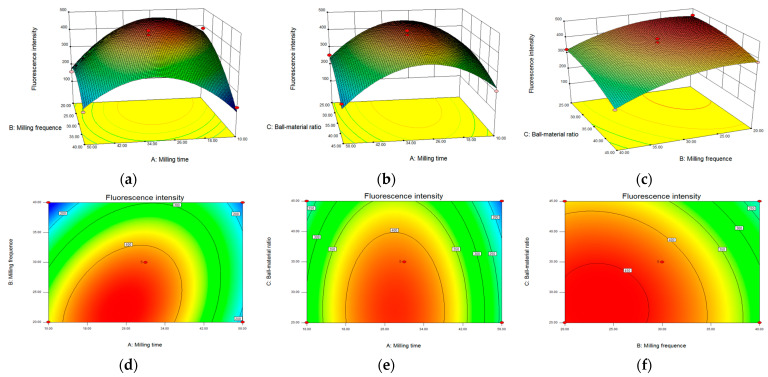
Response surface (**a**–**c**); contour plots (**d**–**e**) of the effect of mechanochemical preparation conditions on the luminescence intensity of MAPbBr_3_/MOF composites.

A response surface analysis was performed on the experimental results described above, and response surfaces and contour plots were obtained for different factors. When the ball–material ratio was constant, the response surface (Figure 2a) and contour lines (Figure 2d) for milling time and frequency were steep and elliptical, indicating a significant interaction between the two variables. When the milling frequency was constant, the degree of curvature of the response surface for milling time and ball–material ratio increased (Figure 2b), and the contour lines were also elliptical (Figure 2e), which also demonstrated the significant interaction between these two factors. However, when the milling time was unchanged, the response curves for milling frequency and ball–material ratio were flat (Figure 2c), and the contour lines were close to circular (Figure 2f), revealing no significant interaction. Further optimization of the above experiments yielded the best preparation conditions for Pb–MOF, with a milling time of 30 min, milling frequency of 30 Hz, and a ball–material ratio of 35:1.

### 2.2. The Structural and Elemental Characterization of Pb–MOF and MAPbBr_3_/MOF Composites

**Figure 3:**
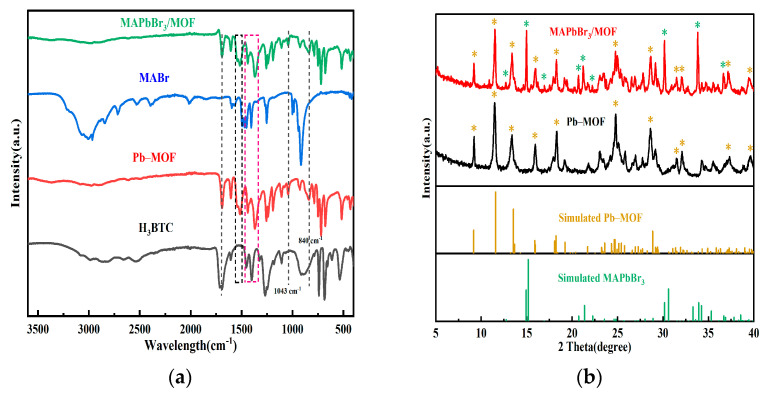
Infrared spectrum (**a**) and XRD patterns (**b**) of Pb–MOF and MAPbBr_3_/MOF composites; the yellow asterisks in subfigure b represent the main simulated characteristic diffraction peaks of Pb–MOF, the green asterisks represent the main simulated characteristic diffraction peaks of MAPbBr_3_.

Infrared spectroscopy and XRD characterization were performed on the Pb–MOF and MAPbBr_3_/MOF composite prepared by mechanochemical methods. Firstly, in comparison with the raw material H_3_BTC, new absorption peaks at 840 cm^−1^, 1043 cm^−1^ and 1510–1550 cm^−1^ were observed in the infrared spectroscopy of Pb–MOF. The symmetric stretching vibration peak of COO^−^ located at 1400–1450 cm^−1^ significantly shifted towards the lower wave number area and the shape of the absorption peak of COO^−^ at approximately 1700 cm^−1^ changed significantly. These changes indicate that the chemical environment of COO^−^ varied after coordination with Pb^2+^. No obvious difference can be observed from the infrared spectra between Pb–MOF and MAPbBr_3_/MOF before and after transformation (Figure 3a), which may be due to the strong absorption of Pb–MOF masking the absorption peak of MAPbBr_3_. The diffraction peaks of the optimized Pb–MOF still matched well with the simulated ones. Comparing the XRD patterns of the MAPbBr_3_/MOF composite with Pb–MOF, it was found that the basic structure of Pb–MOF still remained (the characteristic diffraction peaks were distinct). In addition, obvious new diffraction peaks appeared at 12.7°, 14.9°, 16.9°, 20.7°, 21.3°, 22.2°, 30.1°, 33.9° and 36.6° (Figure 3b), and matched well with the simulated XRD patterns of MAPbBr_3_. The above results indicated the co-presence of MAPbBr_3_ and Pb–MOF in the composite.

**Figure 4:**
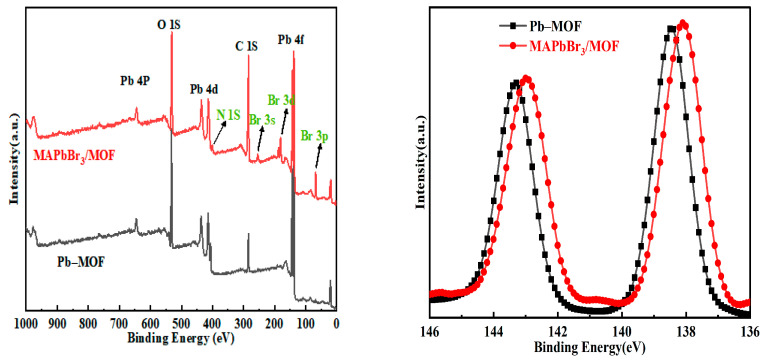
XPS elemental pattern and fitting analysis of Pb–MOF and MAPbBr_3_/MOF composite (**a**) full spectrum; (**b**) Pb 4f spectra.

As shown in the full-range XPS spectra of the Pb–MOF and MAPbBr_3_/MOF composites (Figure 4a), the new signals of Br (Br 3d, 68.11 eV), N (N 1s, 401.45 eV) species appear distinctly in the spectra of MAPbBr_3_/MOF composites. Comparing the MAPbBr_3_/MOF composite with the Pb–MOF, the shift to the lower binding energies (from 138.6 to 138.1 eV) and broadening of the Pb 4f peaks (Figure 4b) suggested a change in coordination chemistry of the Pb atoms. Further peak fitting of the signals for Pb, C, N, O, and Br elements in the MAPbBr_3_/MOF composite and details of all peak-splitting fits are presented in Table S5. This revealed that almost all of the Pb elements (Figure S1a) in the composites were in the Pb–Br signal (138.88 eV, 98%), indicating that the surface Pb was combined with Br. The small amounts of Pb in the elemental state (137.04 eV, 2%) could be attributed to the reduction in lead ions caused by irradiation of X-rays. The signal of the C element (Figure S1b) in the composite at 284.84 eV was attributed to the C–H bond (63%), the signal at 286.2 eV was attributed to the C–O bond (13.62%), and the signal at 288.5 eV was attributed to the COO^−^ group (23.24%). The N element (Figure S1c) in the composite existed only in the form of NH_4_^+^, and its signal peak was at 401.9 eV. The signal of the O element (Figure S1d) in the composite at 531.3 eV was attributed to the C=O group (75%), and the signal at 532.9 eV was attributed to the C–O bond (25%). The Br element (Figure S1e) in the composite existed only in the form of the Pb–Br bond at 68.3 eV. Based on the XPS data (Table 1), the content of MAPbBr_3_ on the surface of MAPbBr_3_/MOF composite was calculated to be 44%.

### 2.3. Stabilities of Pb–MOF and MAPbBr_3_/MOF Composite

**Figure 5:**
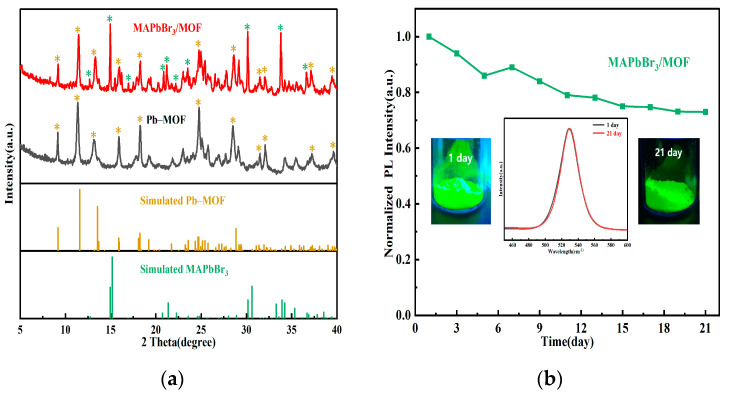
Pb–MOF and MAPbBr_3_/MOF exposed to ambient atmosphere after 21 days (**a**) XRD (**b**) fluorescence stability of MAPbBr_3_/MOF composite; the yellow asterisks in subfigure (**b**) represent the main simulated characteristic diffraction peaks of Pb–MOF, the green asterisks represent the main simulated characteristic diffraction peaks of MAPbBr_3_.

The XRD patterns (Figure 5a) of the Pb–MOF and MAPbBr_3_/MOF composite still matched well with the simulated ones after being exposed to ambient atmosphere for 21 days, which demonstrated that the Pb–MOF and the composite were relatively stable in air. To investigate the emission stability of the MAPbBr_3_/MOF composite, the PL intensity of the MAPbBr_3_/MOF was also monitored. As shown in Figure 5b, the composite preserved 73% of the initial PL intensity with almost unchanged peak shape, suggesting that the emission properties of the composite were maintained [12]. Combining all the experimental results, we infer that the role of the Pb–MOF is mainly to provide a special Pb source and growth environment for MAPbBr_3_, and could prevent deterioration of MAPbBr_3_ in the air.

### 2.4. The Transformation Process of MAPbBr_3_/MOF Composite during Mechanochemical Synthesis

**Figure 6:**
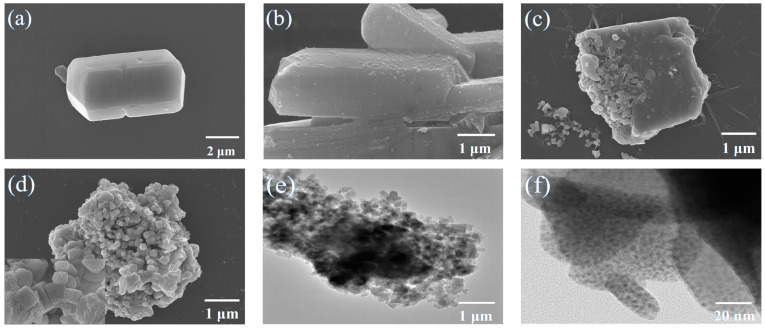
Mechanochemical transformation to form MAPbBr_3_/MOF (**a**) SEM image of Pb–MOF before reaction (**b**) SEM image of MAPbBr_3_/MOF after addition of MABr (**c**) SEM image of MAPbBr_3_/MOF mechanical milling for 3 min (**d**) SEM image of MAPbBr_3_/MOF after mechanical milling; TEM images of MAPbBr_3_/MOF after mechanical milling (**e**) 1 μm (**f**) 20 nm.

The morphological changes in the system at different reaction stages during the preparation of MAPbBr_3_/MOF via the mechanochemical method were analyzed by scanning electron microscopy, which aimed to investigate the transformation process. According to Figure 6, prior to conversion, Pb–MOF was a smooth layered hexahedron of micrometer scale (Figure 6a). Upon addition of MABr and under the action of mechanical milling, the microstructure of the material underwent a transformation: in the initial stage (1 min), MABr diffused into the surface of Pb–MOF and reacted with the Pb ions in the Pb–MOF to form a small amount of MAPbBr_3_, leading to roughening of the surface (consistent with the surface-priority coordination binding reported by Burlakov [18] (Figure 6b). With increased milling time, the bulk Pb–MOF partially crushed under the action of mechanical force, and more Pb ions were released from the Pb–MOF and transformed into MAPbBr_3_, leading to the appearance of two phases, presenting as small particles and large lumps similar to the initial Pb–MOF (Figure 6c). After 5 min of milling, the composite became more uniform, and resulted in hexagonal particles of 200–400 nm (Figure 6d and Figure S2a). Transmission electron microscopy characterization also confirmed the above conversion process (Figure 6e). From Figure 6f, it can be observed that the particle size of MAPbBr_3_ on the surface of the composite was about 5.2 nm after complete conversion (Figure S2b), with an increase in the number of MAPbBr_3_ particles. Although the morphology of the composite was significantly different from the initial Pb–MOF, part of the Pb–MOF still remained and co-presented with MAPbBr_3_ in the composite (certificated by the XRD pattern in Figure 3b). Meanwhile, benefiting from the increased the reaction sites generated from the crush of Pb–MOF during mechanical grinding, more MAPbBr_3_ was generated [11], which explained the significant enhancement in the content of MAPbBr_3_ (calculated from the XPS data in Table 1).

### 2.5. The Universality of the Mechanochemical Method in the Preparation of Organic–Inorganic Hybrid Perovskite/MOF Composites

**Figure 7:**
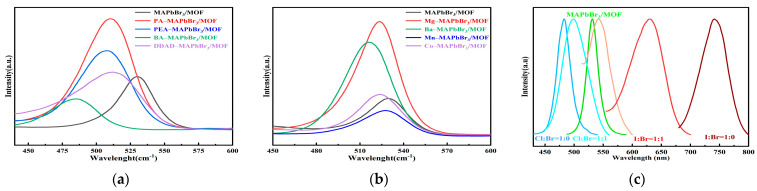
The fluorescence spectra of different doped MAPbBr_3_/MOF composites (**a**) organoamine doped system; (**b**) metal bromide doped system; (**c**) halogen doped system.

Organoamine–MAPbBr_3_/MOF composites with tunable fluorescence properties were synthesized using the above mechanochemical methods by adjusting the species in the organoamines (mixing MABr with PABr, PEABr, BABr, DDADBr). The XRD patterns of the organoamine–MAPbBr_3_/MOF composites illustrated that these organic amine doped composites maintained the basic structure of the MAPbBr_3_/MOF composite, with almost the same characteristic diffraction peaks at 14.9°, 21.3°, 30.1° and 33.9° (Figure S3a), but with multiple peaks at 11.5°. The addition of large organic amines caused a blue shift in the fluorescence peak, with the most pronounced blue shift in BABr, probably because the shorter chain (in contrast to DDADBr) could penetrate into the crystal lattice of MAPbBr_3_ more readily and shear the three-dimensional structure into a quasi-two-dimensional structure [19]. The addition of PABr and PEABr significantly enhanced the fluorescence brightness of the composites (Figure 7a), which may be attributable to benzene ring-like ammonium salts passivating some of the surface defects of the perovskite [20].

Divalent metal ion-doped-MAPbBr_3_/MOF composites were also prepared mechanochemically, with the addition of a suitable amount of metal bromide into the mixture of Pb–MOF and MABr. All the composites displayed the characteristic diffraction peaks of MAPbBr_3_ perovskite (14.9°, 21.3°, 30.1° and 33.9°, Figure S3b), and a small blue shift in the fluorescence emission spectra of the complexes occurred with the addition of the bromide salts, which may possibly result from the lattice contraction of the perovskite unit cell upon alloying [21]. The addition of main-group divalent metal bromides such as MgBr_2_ and BaBr_2_ resulted in a significant increase in the fluorescence intensity of the composites, with an obvious blue shift in Ba–MAPbBr_3_/MOF, while the effect of divalent transition metal bromide salts such as MnBr_2_ and CoBr_2_ was not significant (Figure 7b).

By adjusting the element and content of halogens in MAX, a series of emission-wavelength-tunable organic–inorganic hybrid perovskite/MOF composites were synthesized through the aforementioned mechanochemical approach. Addition of Cl shifted the diffraction peaks (14.9°, 30.1° and 33.9°) of the perovskite towards a higher degree and the addition of I shifted the composites towards a lower degree, which was consistent with literature reports [22]. Multiple diffraction peaks occurred around 11° with the addition of other halogens. Furthermore, once Cl was added, the diffraction peaks of the composites at 11.5° shifted obviously towards the lower degree (Figure S3c). Meanwhile, addition of I eliminated the crystallinity of perovskite. It was observed that as the halogens transitioned from Cl to Br and I, the color of the sample under 365 nm ultraviolet light shifted from deep blue to green and eventually reached deep red (Figure S4). The fluorescence emission peak gradually red-shifted from around 450 nm to almost 800 nm correspondingly (Figure 7c).

These results indicate that the mechanochemical method was expected to have good versatility in the preparation of organic–inorganic hybrid perovskite/MOF composites.

## 3. Materials and Methods

All reagents used in this study were of analytical grade. Lead nitrate (Pb(NO_3_)_2_ purity > 99%) was obtained from Zhongyuan Chemical Reagents (Nanyang, China). Trimesic acid (H_3_BTC purity > 98%) was obtained from Aladdin (Shanghai, China). Anhydrous ethanol (purity > 99.7%) and anhydrous ether (CH_3_OCH_3_ purity > 99.7%) were obtained from Hongyuan Chemical Reagents (Hefei, China). Hydrogen bromide (HBr 40% aqueous solution) and hydrogen iodide (HI 57% aqueous solution) were obtained from Aladdin (Shanghai, China). Hydrogen chloride (HCl 37%) was obtained from Aladdin (Shanghai, China). Methylamine (CH_3_NH_2_ 30–33 wt% methanol solution) was obtained from Fuchen Chemical Reagents (Tianjing, China). Benzylamine (PA purity > 99%) was obtained from Aladdin (Shanghai, China). β-phenylethylamine (PEA purity > 98%) was obtained from Aladdin (Shanghai, China). n-Butylamine (BA purity > 99%) was obtained from Aladdin (Shanghai, China). 1,10-decanediammonium dibromide (DDADBr purity > 98%) was obtained from Aladdin (Shanghai, China). Magnesium bromide, barium bromide, manganese bromide and cobalt bromide were obtained from Aladdin (Shanghai, China).

### 3.1. Preparation of Pb–MOF

Preparation of Pb–MOF using the mechanochemical method [23]: 662.46 mg Pb(NO_3_)_2_ and 420.48 mg H_3_BTC were loaded into a ball-milling tank. The assistant solvents and the ball milling parameters such as milling time, milling frequency, and the ball–material ratio were adjusted as needed. The product was purified by means of centrifugation, alternately with anhydrous ethanol and distilled water before being dried and stored.

### 3.2. Perovskite/MOF Composites

Preparation of methylamine hydrohalite (MAX): Following the procedure reported previously [24], the hydrogen halide solution (0.5 M) was added dropwise to the methylamine solution (0.4 M, 5 mL) and the reaction continued for 3 h under constantly stirring in an ice-bath until crystallization occurred. The obtained MAX (MACl, MABr, MAI) crystals were dissolved in anhydrous ethanol and then extracted with diethyl ether, and the process repeated three times. The resulting product was dried under vacuum at 60 °C for 6 h and stored for later use. The PABr, PEABr, BABr were prepared in the same way as MAX, except that the methylamine solution was replaced with PA, PEA and BA, respectively.

Preparation of MAPbBr_3_/MOF using mechanochemical method: 1 g purified Pb–MOF was poured into a ball-milling tank, then 100 mg MABr was added and the mixture was milled for 5 min to accomplish the transformation. The final products were obtained and classified as MAPbBr_3_/MOF.

Preparation of organic ammonium–MAPbBr_3_/MOF using the mechanochemical method: 1 g purified Pb–MOF was poured into a ball-milling tank, then 80 mg MABr and 20 mg organic ammonium (PABr, PEABr, BABr, DDADBr) were added and the mixture was milled for 5 min to accomplish the transformation. The final products were obtained and classified as PA–MAPbBr_3_/MOF, PEA–MAPbBr_3_/MOF, BA–MAPbBr_3_/MOF, DDAD–MAPbBr_3_/MOF.

Preparation of metal–MAPbBr_3_/MOF using the mechanochemical method: The dosage and mechanical grinding conditions for Pb–MOF and MABr were the same as those for MAPbBr_3_/MOF, with the exception that 200 mg of divalent metal bromide (MgBr_2_, BaBr_2_, MnBr_2_, CoBr_2_) was added to obtain the metal-doped system of Mg–MAPbBr_3_/MOF, Ba–MAPbBr_3_/MOF, Mn–MAPbBr_3_/MOF, Co–MAPbBr_3_/MOF.

Preparation of MAPbX_3_/MOF using the mechanochemical method: 1 g purified Pb–MOF was poured into a ball-milling tank, then a mixture of 100 mg of MACl, MABr and MAI were added, and the mixture was milled for 5 min to accomplish the transformation. The final products were obtained and classified as MAPbX_3_/MOF.

### 3.3. Characterization

Fourier transform infrared spectroscopy (FTIR-650, Shanghai Noding Instrument Equipment, Shanghai, China, scanning wave number 400–4000 cm^−1^); XRD powder diffractometer (Empyrean Panaco, Almelo, The Netherlands, radiation source Cu Kα, λ = 0.154056 nm, operating voltage 40 kV, current 150 mA, measurement range 5–60°, scanning speed 5° min^−1^); X-ray electron spectroscopy (XPS) (OXFORD Xplore, Oxford, UK, measured with Al Kα rays as the excitation source (hν = 1486.6 eV) and C1s binding energy 284.8 eV as the reference correction); Fluorescence spectrophotometer (Shimadzu CrayEclipse, Tokyo, Japan, excitation wavelength 365 nm, emission wavelength scanning interval 450–600 nm); Scanning electron microscope (SEM) (ZEISS Gemini 300, Oberkochen, Germany); Transmission electron microscope (TEM) (Thermo Fisher FEI Talos F200X, Wyman Street, Waltham, MA, USA).

## 4. Conclusions

In summary, we have developed a green and efficient approach to prepare environmentally stable organic–inorganic hybrid perovskite luminescent materials using Pb–MOF as a particular lead source through a mechanochemical method. Optimal performance of MAPbBr_3_/MOF was obtained from Pb–MOF prepared under solvent-free conditions, with a milling time of 30 min, milling frequency of 30 Hz and ball–material of 35:1. Benefiting from the increased reaction sites generated from the crush of Pb–MOF during mechanical grinding, more MAPbBr_3_ was generated with the particle size of approximately 5.2 nm, although the morphology of the composite was significantly different from the initial Pb–MOF. It was also confirmed that the mechanochemical method had a good universality in preparing organic–inorganic hybrid perovskite/MOF composites.

## Figures and Tables

**Table 1:** XPS analysis of MAPbBr_3_/MOF composite element.

Sample	Elements	Peak	Height	Atomic %	MAPbBr_3_ %
	Br 3d	67.83	18,687.72	4.52	
	Pb 4f	138.1	169,444.58	3.39	
MAPbBr_3_/MOF	C 1s	284.21	90,371.65	63.07	44.4
	N 1s	401.22	8541.33	3.69	
	O 1s	531.03	96,104.52	25.33	

## Data Availability

The data presented in this study are available on request from the corresponding author. The data are not publicly available due to privacy restrictions.

## Supplementary Materials

The following supporting information can be downloaded at: https://www.mdpi.com/article/10.3390/molecules28135021/s1, Table S1: Table of factors and levels in factorial tests; Table S2: Response surface design and fluorescence intensity results; Table S3: Analysis of variance of the response surface regression model of mechanochemical conditions on the luminescence intensity of MAPbBr_3_/MOF composites; Table S4: Systematic analysis of regression equation error; Table S5: Table of peak positions for XPS split peak fitting; Figure S1: Fitting analysis of XPS elemental pattern of MAPbBr_3_/MOF composites (a) fit plot of Pb; (b) fit plot of C; (c) fit plot of N; (d) fit plot of O; (e) fit plot of Br; Figure S2: Particle size distribution of MAPbBr_3_/MOF composites (a) MAPbBr_3_/MOF; (b) MAPbBr_3_; Figure S3: XRD patterns of perovskite/MOF with (a) different ammonium systems; (b) different metal bromide systems; (c) different halogen systems; Figure S4: Optical images of organic–inorganic hybrid perovskite/MOF composites with different halogen.
