# Supplementary material for: Synthesis of Organic–Inorganic Hybrid Perovskite/MOF Composites from Pb–MOF Using a Mechanochemical Method
Source: Molecules. 2023 Jun 27;28(13):5021. doi: 10.3390/molecules28135021 (PMC10343487; doi:10.3390/molecules28135021)
Supplement: Supplementary file 1 [file molecules-28-05021-s001.zip › molecules-2463691-supplementary.pdf]

Supporting Information

Table S1. Table of factors and levels in factorial tests

	Factor	level		
		-1	0	1
A	Milling time/min	10.00	30.00	50.00
B	Milling frequency/Hz	20.00	30.00	40.00
C	ball-material ratio	25.00	35.00	45.00

Table S2. Response surface design and fluorescence intensity results

**Std**	**Run**	**Factor 1 A.Milling time(min)**	**Factor 2 B.Milling frequency(Hz)**	**Factor 3 C.Ball-material ratio**	**Response1 luminescent intensity**
3	1	50.00	30.00	45.00	175
6	2	10.00	40.00	35.00	123
4	3	30.00	30.00	35.00	454
7	4	10.00	30.00	25.00	279
2	5	10.00	20.00	35.00	386
8	6	50.00	40.00	35.00	136
11	7	50.00	20.00	35.00	157
5	8	30.00	30.00	35.00	430
13	9	50.00	30.00	25.00	254
14	10	30.00	30.00	35.00	426
17	11	30.00	40.00	25.00	325
16	12	30.00	30.00	35.00	395
10	13	30.00	20.00	45.00	380
9	14	30.00	20.00	25.00	475
12	15	30.00	40.00	45.00	214
1	16	10.00	30.00	45.00	207
15	17	30.00	30.00	35.00	420

Table S3. Analysis of variance of the response surface regression model of mechanochemical conditions on the luminescence intensity of $MAPbBr_3$/MOF composites

**Source**	**Sum of Squares**	**df**	**Mean Squares**	**F Value**	**P-Value Prob＞F**	
Model	2.26E+005	9	25102.35	34.13	＜0.0001	significant
A-time	9316.13	1	9316.13	12.67	0.0092	
B-Frequencies	42340.50	1	42340.50	57.58	0.0001	
C-ball material ratios	14365.13	1	14365.13	19.53	0.0031	
AB	14641.00	1	14641.00	19.91	0.0029	
AC	12.25	1	12.25	0.017	0.9009	
BC	289.00	1	289.00	0.39	0.5506	
A^2	1.215E+005	1	1.215E+005	165.23	＜0.0001	
B^2	12563.75	1	12563.75	17.08	0.0044	
C^2	2929.01	1	2929.01	3.98	0.0862	
Residual	5147.75	7	735.39			
Lack of Fit	3355.75	3	1118.58	2.50	0.1988	not significant
Pure Error	1792.00	4	448.00			
Cor Total	2.311E+005	16				

Table S4. Systematic analysis of regression equation error

Std. Dev.	27.12	R^2	0.9777
Mean	306.94	Adj-R^2	0.9491
C. V. %	8.83	Pred-R^2	0.7555
PRESS	56492.00	Adeq-Precision	16.992

Table S5. Table of peak positions for XPS split peak fitting

Name	Peak (BE)	FWHM (eV)	Area(P)C PS.eV
C1s	284.84	1.25	58,907.70
C1s	286.24	1.27	12,700.09
C1s	288.52	1.33	21,669.93
O1s	531.36	1.56	121,899.16
O1s	532.98	1.56	40,629.19
N1s	401.91	1.30	3,086.60
Br3d5	68.32	1.08	7,751. 98
Br3d3	69 .36	1.08	5,352.56
Pb4f7	137.04	1.50	3581.05
Pb4f5	142.19	1.50	2721.60
Pb4f7	138.88	1.25	153875.93
Pb4f5	143.76	1.25	116945.70

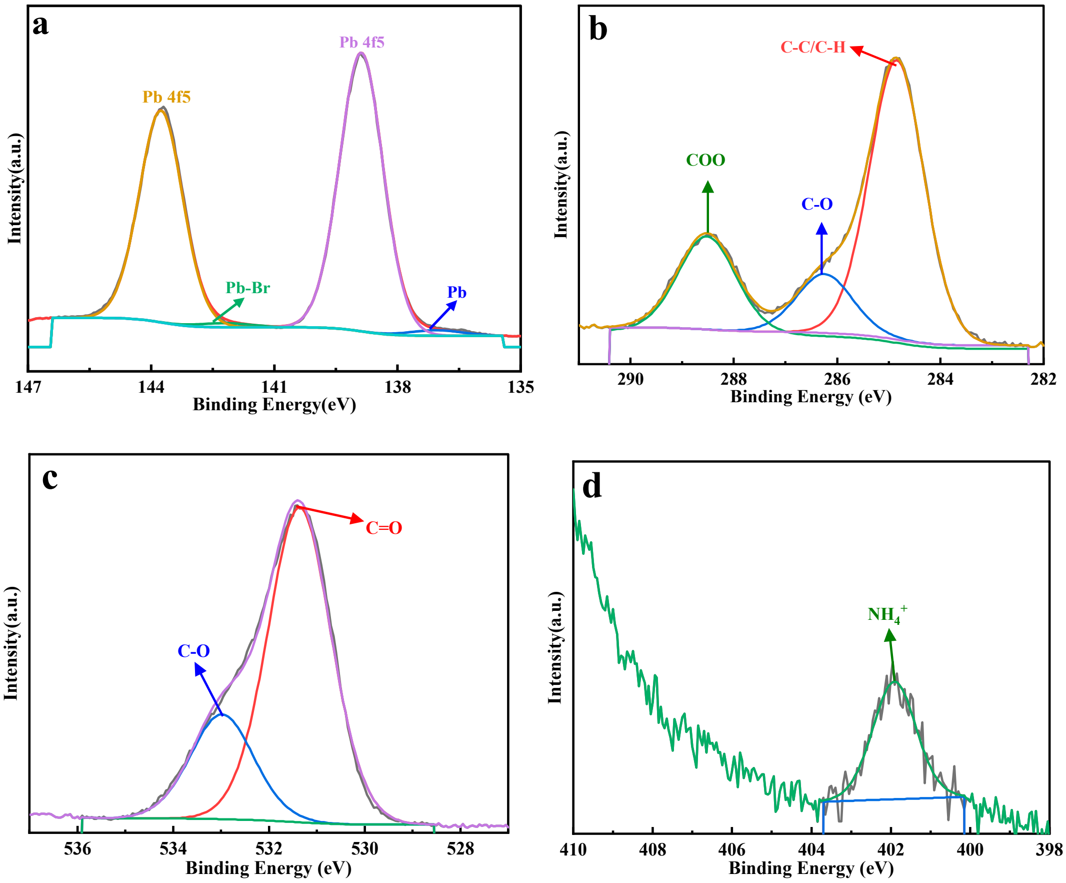

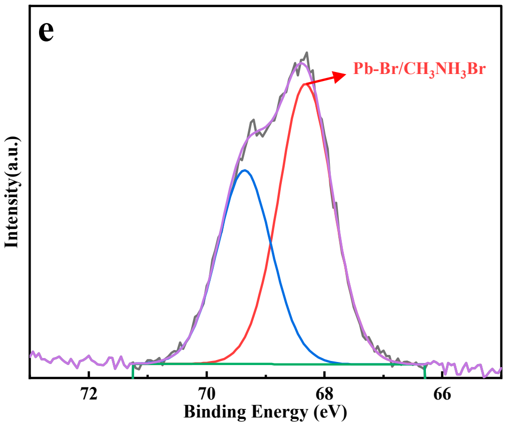

Figure S1. Fitting analysis of XPS elemental pattern of MAPbBr3/MOF composites (a) fit plot of Pb; (b) fit plot of C; (c) fit plot of N; (d) fit plot of O; (e) fit plot of Br

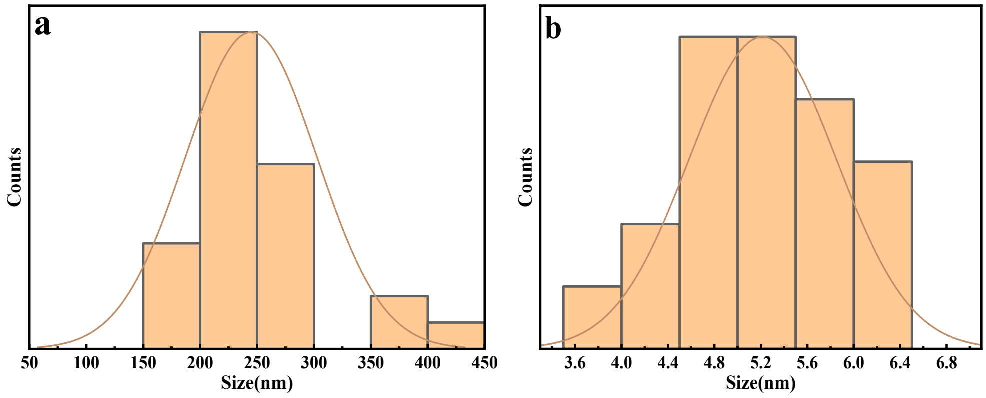

Figure S2. Particle size distribution of MAPbBr3/MOF composites (a) MAPbBr3/MOF; (b) MAPbBr3

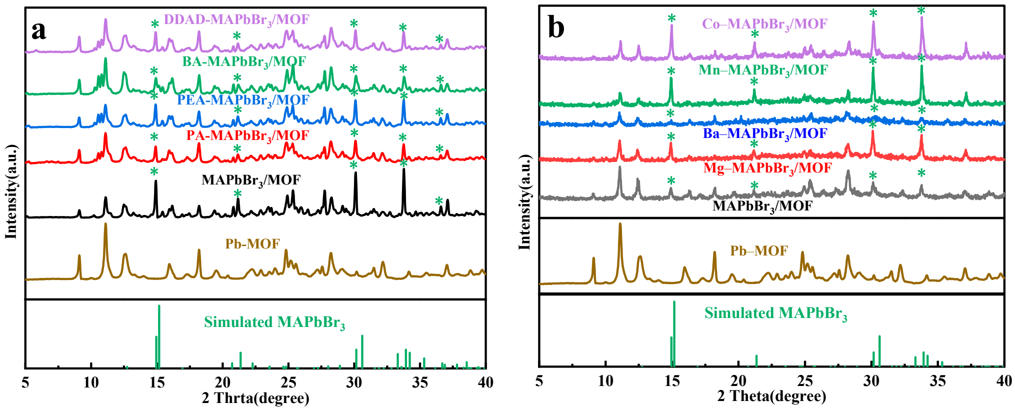

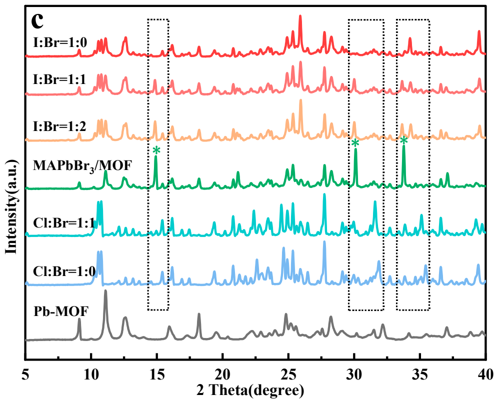

Figure S3. XRD patterns of perovskite/MOF with (a) different ammonium systems; (b) different metal bromide systems; (c) different halogen systems

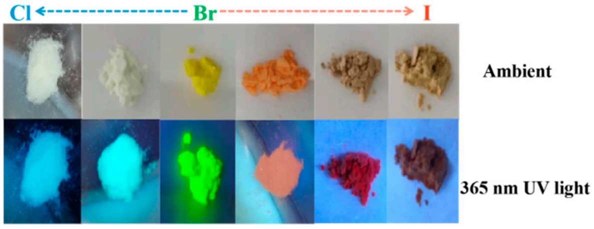

Figure S4. Optical images of organic-inorganic hybrid perovskite/MOF composites with different halogen
